# Assessment of Malawi’s success in child mortality reduction through the lens of the Catalytic Initiative Integrated Health Systems Strengthening programme: Retrospective evaluation

**DOI:** 10.7189/jogh.05.020412

**Published:** 2015-12

**Authors:** Tanya Doherty, Wanga Zembe, Nobubelo Ngandu, Mary Kinney, Samuel Manda, Donela Besada, Debra Jackson, Karen Daniels, Sarah Rohde, Wim van Damme, Kate Kerber, Emmanuelle Daviaud, Igor Rudan, Maria Muniz, Nicholas P Oliphant, Texas Zamasiya, Jon Rohde, David Sanders

**Affiliations:** 1 Health Systems Research Unit, South African Medical Research Council, Cape Town, South Africa; 2 School of Public Health, University of the Western Cape, Cape Town, South Africa; 3 School of Public Health, University of the Witwatersrand, Johannesburg, South Africa; 4 Saving Newborn Lives/Save the Children, Cape Town, South Africa; 5 Biostatistics Research Unit, South African Medical Research Council, Pretoria, South Africa; 6 School of Mathematics, Statistics and Computer Science, University of Kwazulu-Natal, Durban, South Africa; 7 UNICEF, United Nations Plaza, New York, NY, USA; 8 Institute of Tropical Medicine, Antwerp, Belgium; 9 Centre for Global Health Research and Global Health Academy, University of Edinburgh Medical School, Edinburgh, Scotland, UK; 10 UNICEF, Lilongwe, Malawi; 11 School of Child and Adolescent Health, Faculty of Health Sciences, University of Cape Town, Rondebosch, South Africa

## Abstract

**Background:**

Malawi is estimated to have achieved its Millennium Development Goal (MDG) 4 target. This paper explores factors influencing progress in child survival in Malawi including coverage of interventions and the role of key national policies.

**Methods:**

We performed a retrospective evaluation of the Catalytic Initiative (CI) programme of support (2007–2013). We developed estimates of child mortality using four population household surveys undertaken between 2000 and 2010. We recalculated coverage indicators for high impact child health interventions and documented child health programmes and policies. The Lives Saved Tool (LiST) was used to estimate child lives saved in 2013.

**Results:**

The mortality rate in children under 5 years decreased rapidly in the 10 CI districts from 219 deaths per 1000 live births (95% confidence interval (CI) 189 to 249) in the period 1991–1995 to 119 deaths (95% CI 105 to 132) in the period 2006–2010. Coverage for all indicators except vitamin A supplementation increased in the 10 CI districts across the time period 2000 to 2013. The LiST analysis estimates that there were 10 800 child deaths averted in the 10 CI districts in 2013, primarily attributable to the introduction of the pneumococcal vaccine (24%) and increased household coverage of insecticide–treated bednets (19%). These improvements have taken place within a context of investment in child health policies and scale up of integrated community case management of childhood illnesses.

**Conclusions:**

Malawi provides a strong example for countries in sub–Saharan Africa of how high impact child health interventions implemented within a decentralised health system with an established community–based delivery platform, can lead to significant reductions in child mortality.

Since 2010, Malawi has been on track to reach Millennium Development Goal (MDG) 4 and is one of the first countries in sub–Saharan Africa to have reached the target [1], despite reporting one of the lowest gross national incomes per capita in the world [2]. According to the UN Inter–Agency Group for Child Mortality Estimation, under–5 mortality has declined steadily from 245 to 68 deaths per 1000 live births between 1990 and 2013 [3].

Malawi showed commitment to accelerating child survival and development through the establishment of the Accelerated Child Survival and Development policy in 2006 which was implemented through the Integrated Management of Childhood Illness (IMCI) five year strategic plan (2006–2011) and the Strategic Plan for Child Survival in 2007. The strategy aimed to reduce childhood morbidity and mortality by two–thirds between 2000 and 2015, and it focused on the scaling up of high impact interventions including integrated community case management of childhood illnesses and newborn care (iCCM) [4].

From 2008 iCCM was scaled up nationally under the coordination of the Ministry of Health with the support of partners in different districts including UNICEF, WHO, Save the Children, and others. UNICEF worked as an implementing partner in ten of twenty–eight districts throughout the country through the Catalytic Initiative (CI) Integrated Health Systems Strengthening (IHSS) programme (**Figure 1**) whilst other partners provided similar support in the remainder of the country. In 2013, an estimated 41 000 under–5 deaths occurred nationally with approximately 47% in the ten CI focus districts [3].

**Figure 1:**
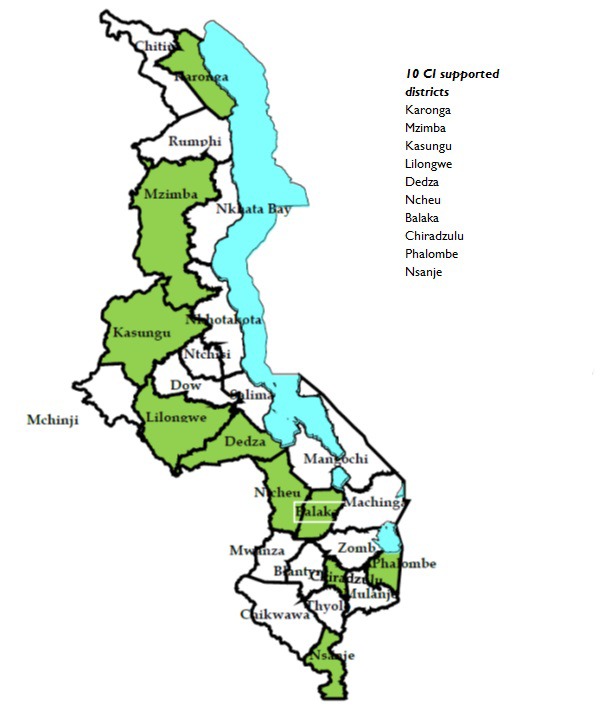
Map of Malawi showing 10 Catalytic Initiative districts (shaded in green).

The CI/IHSS programme was established by UNICEF with joint funding from the Department of Foreign Affairs, Trade and Development Canada (DFATD) in late 2007 with the main aim of assisting low and middle–income countries in Central, West and Southern Africa with high maternal and child mortality rates, including Malawi, to scale up services to children and pregnant women [5]. The CI programme had a strong health systems strengthening focus through training of front line health workers, provision of drugs and supplies, support for supervision and development of monitoring and evaluation systems [6]. In the initial period the CI programme in Malawi supported mainly preventive interventions including provision of vitamin A supplementation, immunisations, counselling on infant and young child feeding as well as training almost 4000 nurses in Integrated Management of Childhood Illnesses (IMCI). Following the Ministry of Health decision to scale up iCCM, the focus was on training and equipping of community health workers called health surveillance assistants (HSAs) to deliver iCCM services for treatment of malaria, pneumonia and diarrhoea [7]. As one of six countries participating in the CI programme, Malawi received a total of US$ 19.4 million from DFATD (US$ 11.5) and UNICEF (US$ 7.9) over the grant period 2007– 2013 [8].

HSAs have played a central role in the delivery of health services in Malawi since the 1960s, delivering an increasingly broad array of services at the community level [9–11]. Initially operating as environmental health outreach assistants concentrating on water and sanitation; since 1995 HSAs have been formally recruited and salaried by the Ministry of Health. Required to have grade 10 (junior certificate of education) to qualify, HSAs receive 12 weeks of general training, and since 2008, an additional 6 days of specific training on iCCM. They split their working week between the village clinic (situated in hard–to–reach areas >5km from the nearest health centre) in which they spend 2–3 days per week, community–based outreach work and assisting in fixed health facilities. In 2011 there were over 10 000 HSAs in the country of which 3800 had been trained in iCCM with just over 1000 of these situated in the 10 districts where CI efforts were focused (**Figure 1**) [10]. Small–scale evaluations during the early period of iCCM implementation (2009–2011) revealed high demand for HSA services [10] and quality of care similar to that provided by nurses in first–level facilities [12]. An evaluation of changes in newborn survival identified areas for further work, including the integration of neonatal sepsis management into iCCM [13]. However, no evaluation has assessed the broader impact of child survival strategies, particularly iCCM, on child health indicators in Malawi. This paper explores factors influencing progress in child survival including coverage of interventions, the role of key national policies and impact of coverage change on under–5 deaths averted using data from an evaluation of the CI programme of support in Malawi.

## METHODS

### Study design and setting

The analyses undertaken were part of a multi–country retrospective evaluation of the CI programme. The selection of the 10 CI districts for UNICEF support was undertaken jointly by UNICEF and the Ministry of Health (**Figure 1**). The selected districts reported higher rates of maternal, newborn and child mortality in 2006 [14] compared to national mortality and included remote areas with limited health care access. The CI grant supported both facility and community–based interventions including preventive and curative services (**Box 1**). This evaluation compared average annual change (AAC) in coverage for key indicators in the 10 CI districts before the CI support began (2000–2006) and during the period of implementation (2007–2013).

Box 1 **Interventions supported in Malawi through the Catalytic Initiative funding**

**Expanded Programme on Immunisation:**

• Catch up immunisation through child health days

• Vitamin A supplementation

**Health system strengthening of the health surveillance assistant (HAS) platform** (particularly related to integrated community case management (iCCM) of malaria, pneumonia and diarrhoea):

• Communication and social mobilisation on iCCM (through job aids)

• Recruitment, selection and training of HSAs

• Basic supplies for HSAs (drug box, bicycles, motorcycles for supervision)

• Supervision (quarterly mentorship and review meetings on iCCM)

• M&E (support to M&E officer at IMCI unit)

• Review of health surveillance curriculae to include new competencies **Renovation of three training centers:**

• Purchased sachets of oral rehydration salts (ORS) and zinc tablets, cotrimoxazole, sulfadoxine–pyrimethamine and artemisinin–combination therapies (ACTs) for village clinics

**Integrated Management of Childhood Illnesses (IMCI):**

• Training of nurses and clinicians in IMCI

**Malaria prevention:**

• Supply and distribution of ITNs for pregnant women and children under five years

**Health promotion**

**Infant and young child feeding:**

• Promotion of early initiation and exclusive breastfeeding for six months

• Screening for severe and acute malnutrition

**WASH:**

• Education on safe water, sanitation and hygiene

### Data sources

We used birth and death history data collected from women aged 15 to 49 years in nationally representative surveys: namely the 2000 Demographic and Health Survey (DHS), 2004 DHS, 2006 Multiple Indicator Cluster Survey (MICS), and the 2010 DHS to calculate under–5 mortality. The surveys covered 14 213, 13 664, 30 553, and 24 825 households respectively.

For analysis of intervention coverage we used standard indicator definitions [15] for 11 interventions targeted by the CI for tracking progress towards MDG 4 (**Table 1**). We also captured coverage change for other maternal and contextual indicators. Surveys included in the analysis of intervention coverage were the 2000 DHS, 2006 MICS, 2010 DHS and the 2013 Lot Quality Assurance Survey (LQAS) which sampled in the 10 CI districts only [16,17]. The 2004 DHS did not include disaggregated data for all of the CI districts; therefore it was excluded from the coverage analysis (Section A in **Online Supplementary Document(Online Supplementary Document)**). All surveys provided cross–sectional data on intervention coverage in their respective years. Full survey data sets with district sampling weights were used for the analysis. For further details on the surveys included in the analysis see Table s1 in **Online Supplementary Document(Online Supplementary Document)**. Adjustments were made to align indicator definitions across the DHS, MICS and LQAS surveys (Section B in **Online Supplementary Document(Online Supplementary Document)**).

**Table 1:** Summary of indicator coverage change in the 10 Catalytic Initiative–focus districts

Indicator	Malawi (10 CI districts)			Average annual change pre–CI (2000–2006: period 1; % per year with confidence intervals)	Average annual change during CI (2006–2013: period 2; % per year with confidence intervals)	Direction of change between period 1 and period 2‡
DHS 2000 (pre–CI) % (95%CI)	MICS 2006 (baseline) % (95%CI)	LQAS 2013 (endline) % (95%CI)
Tetanus toxoid vaccination of pregnant women (at least 2 doses)	58 (56 to 61)	72 (70 to 74)	72 (69 to 75)	3.6 (3.1 to 4.1)	0.0 (N/A)	↓
IPTp	28 (26 to 31)	48 (46 to 51)	84 (82 to 87)	8.9 (8.1 to 9.8)	7.9 (7.5 to 8.4)	→
Early breastfeeding	68 (66 to 71)	53 (51 to 56)	75 (72 to 79)	–4.1 (–4.6 to –0.2)	5.0 (4.4 to 5.6)	↑
Exclusive breastfeeding	38 (33 to 43)	55 (50 to 60)	61 (57 to 64)	6.1 (4.8 to 7.5)	1.5 (0.5 to 2.4)	↓
Vitamin A supplementation*	79 (76 to 82)	75 (71 to 78)	56 (52 to 59)	–0.8 (–1.1 to –0.6)	–4.2 (–4.9 to –3.4)	↓
Measles immunisation	80 (77 to 84)	81 (78 to 84)	87 (84 to 89)	0.2 (to 0.2 to 0.6)	1.0 (0.6 to 1.4)	↑
DPT3 immunisation	82 (79 to 85)	86 (83 to 88)	88 (86 to 91)	0.8 (0.4 to 1.2)	0.3 (0.0 to 0.7)	→
Care–seeking of suspected pneumonia	26 (23 to 29)	52 (46 to 58)	78 (75 to 81)	11.5 (10.1 to 12.9)	5.8 (4.9 to 6.7)	↓
ACTs for malaria	None†	0.08 (–0.01 to 0.17)	53 (49 to 56)	–	92.8 (79.9 to 105.7)	
ITNs	2 (2 to 3)	25 (23 to 26)	46 (42 to 49)	42.1 (39.3 to 44.8)	8.7 (7.7 to 9.7)	↓
ORS use	47 (42 to 52)	50 (46 to 53)	61 (57 to 64)	1.0 (0.0 to 2.0)	2.8(2.0 to 3.7)	↑

IPTp – intermittent preventive treatment of malaria for pregnant women, ITNs – percent of children <5 who slept under an Insecticide Treated Net the previous night, DPT – diphtheria, pertussis and tetanus, ACTs – Artemisinin–combination therapies, ORS – Percentage of children <5 with diarrhoea in the last 2 weeks who received oral rehydration salts

*Amongst children aged 12–23 moths.

†ACTs were only introduced as first line malaria treatment in 2008.

‡Arrows in the last column indicate whether average annual change in coverage decreased, was stable or increased between period 1 and period 2: ↓ - decrease in AAC between pre–CI (period 1) and during CI (period 2); → – stable AAC between pre–CI (period 1) and during CI (period 2), ↑ – increase in AAC between pre–CI (period 1) and during CI (period 2).

**Figure 2:**
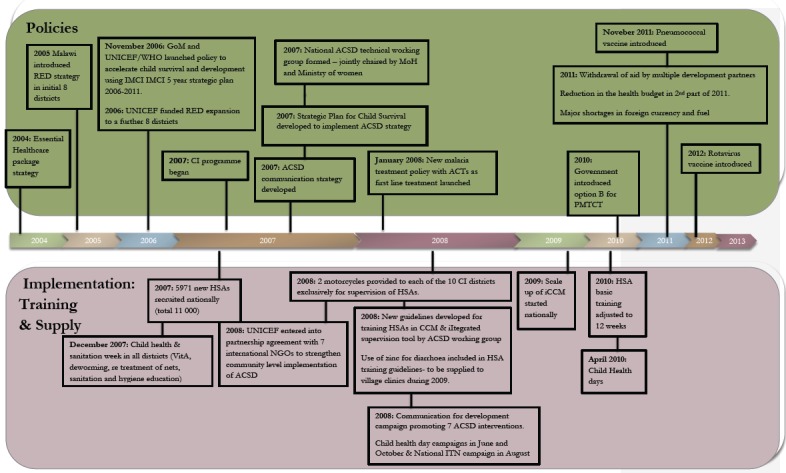
Major policy changes and programmatic activities related to child survival in Malawi (Catalytic Initiative districts and nationally), 2004 – 2012. RED – Reach Every District Strategy; ACSD – Accelerated Child Survival and Development policy; GoM – Government of Malawi; IMCI – Integrated Management of Childhood Illness; MoH – Ministry of Health; CI – Catalytic Initiative; ACTs – Artemisinin–combination therapies for the treatment of malaria; HSA – Health surveillance assistant; NGO – Non-governmental organisation; ITN – insecticide-treated bed net.

Contextual information about child health policies, CI implementation and other relevant child health programmes was obtained through a desk review of documents and databases obtained during a 10–day country visit (August 2013). The information gathered from these sources was used to compile a policy and programme timeline (**Figure 2****)**. For further details on the contextual analysis see Panel s1 in **Online Supplementary Document(Online Supplementary Document)**.

### Statistical analysis

We used a direct method for estimating under–5 mortality based on the synthetic cohort approach [18,19]. Under this concept, age–specific mortality probabilities for narrow age ranges and defined periods are calculated using death events and exposures. These probabilities are combined to compute the probability that a child has not died before reaching age 5 years [19]. Five–periods were used beginning with five years before the survey, and survival probabilities were calculated over age ranges; 0, 1–2, 3–5, 6–11, 12–23, 24–35, 36–47, 48–59 months as recommended by DHS (Section C in **Online Supplementary Document(Online Supplementary Document)**) [19]. The standard errors for the computed mortality estimates were obtained using the Jackknife variance estimation, a repeated sampling method [18]. A series of mortality estimates were obtained by deleting and replacing each primary sampling unit; this produced a sample of under–5 estimates, from which the variance was computed in turn. We also estimated the AAC in mortality using mortality estimates for the periods 1991–1995 and 2006–2010 (Section C in **Online Supplementary Document(Online Supplementary Document)**).

For analysis of intervention coverage, the 10 CI districts were treated as one stratum. We re–calculated all relevant coverage indicators from each survey data set in order to obtain the confidence intervals around the estimates. We then assessed whether there was a significant difference in the AAC in coverage for 11 indicators between the pre–CI period (2000–2006) and the CI implementation period (2006–2013) for the 10 CI districts. The 95% confidence intervals (95% CI) around the AAC on the log scale were based on standard deviations calculated using the delta method for the log function of a proportion.

The 95% confidence intervals were used to assess whether the changes were significantly different between pre–CI and CI periods. In order to check the hypothesis that the simultaneous national scale up of iCCM would result in similar coverage change between CI and non–CI districts (supported by other partners), we calculated AAC in intervention coverage in CI and non–CI districts between 2000 and 2010 (data for the non–CI districts was not collected in the 2013 LQAS).

To assess the contribution of iCCM by HSAs, data relating to care and treatment sought for fever, suspected pneumonia and diarrhoea by place of treatment were extracted from the available household surveys. The 2006 MICS only collected data on place of treatment for suspected pneumonia but not for diarrhoea or fever [20] and it was therefore not included in this analysis.

The sampling design of the household surveys such as regional and rural/urban stratification, clustering at enumeration areas and sampling weights (due to non–proportional sampling) were taken into account. We used Stata (version 12) for these analyses [21].

An attempt to quantify the association between change in contextual factors and intervention coverage with change in under–5 mortality in a multivariate analysis did not yield meaningful results due to the limited number of data points for macroeconomic contextual variables (Section D in **Online Supplementary Document(Online Supplementary Document)**).

We used the Lives Saved Tool (LiST) [22] to forecast child mortality (rates and deaths) in the 10 CI districts in 2013 on the basis of the above measured baseline values of mortality in children younger than 5 years for the period 2006–2010 (Section E in **Online Supplementary Document(Online Supplementary Document)**) and interpolated changes in coverage from the MICS 2006, DHS 2010 and LQAS 2013. We present the estimates of lives saved in 2013, relative to 2008 when CI implementation began, and used the LiST model to investigate the extent to which the declines in child mortality could be attributed to changes in intervention coverage. We also considered the proportion of deaths averted between 2000 and 2008 using our measured baseline mortality and coverage data from the DHS 2000, MICS 2006 and DHS 2010 to compare results between pre–CI and CI periods. The LiST modelling methods have been widely published, including discussion of the limitations which are particularly related to the lack of population–based coverage data for certain key interventions [22–24].

Specific input values used in this LiST application are available in Table s6 in **Online Supplementary Document(Online Supplementary Document)**. The analysis was done with the computer programme Spectrum/ Lives Saved Tool, version 5.04. The study received ethical approval from the ethics committee of the South African Medical Research Council (EC021–9/2012).

## RESULTS

**Figure 3:**
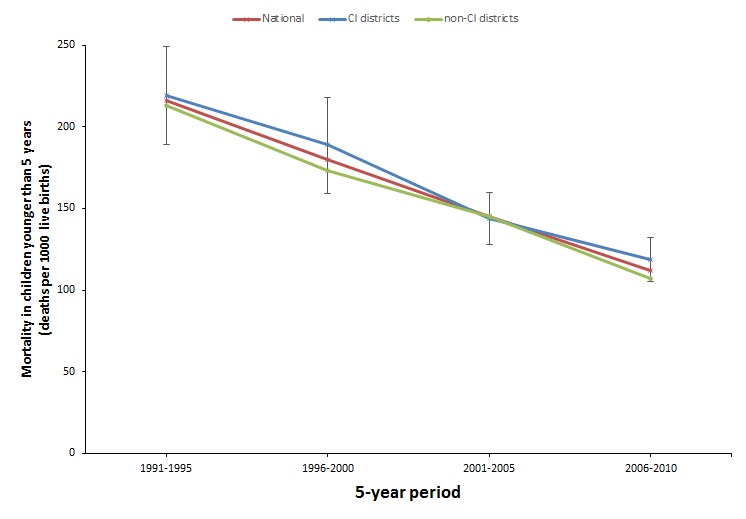
Under–5 mortality rates from 1991 to 2010 in Malawi. Data are from analysis of the 2010 national DHS survey in Malawi. Vertical lines show 95% CIs for survival probabilities for the Catalytic Initiative district estimates. Dates on the x–axis represent the 5–year periods preceding the 2010 Malawi DHS.

The mortality rate in children younger than 5 years decreased rapidly in the 10 CI districts from 219 child deaths per 1000 live births (95% CI 189–249) in the period 1991–1995 to 119 child deaths per 1000 live births in the period 2006–2010 (105–132) with an average annual change of –4.1%. The mortality decline was similar nationally and in the non–CI districts (**Figure 3**).

Improvements in the coverage of interventions relevant to child survival in the 10 CI districts across the time period 2000 to 2013 were found with regard to all indicators except vitamin A supplementation (**Table 1**). For certain indicators the AAC was higher in the CI period compared to the pre–CI period (early breastfeeding (initiation within 1 hour), measles immunisation and oral rehydration salts (ORS) use) whilst for others (DPT3 immunisation and intermittent preventive treatment of malaria for pregnant women (IPTp) the coverage increases were maintained at the same rate as the pre–CI period. The AAC decreased during the CI period for tetanus toxoid vaccination for pregnant women, exclusive breastfeeding, vitamin A supplementation, care–seeking for pneumonia and insecticide–treated bednets (ITNs) (**Table 1**). While AAC with regard to care seeking for pneumonia and ITNs was not as large during the CI period, both the pre–CI and CI periods reflected gains in coverage.

The AAC in coverage for the examined high impact child health interventions in the CI districts compared to the non CI districts between 2000 and 2010 showed no statistically significant difference for any of the indicators (Table s7 in **Online Supplementary Document(Online Supplementary Document)**), meaning that although coverage levels differed by intervention and by district, the AAC was consistent across the country for each intervention examined.

**Figure 4:**
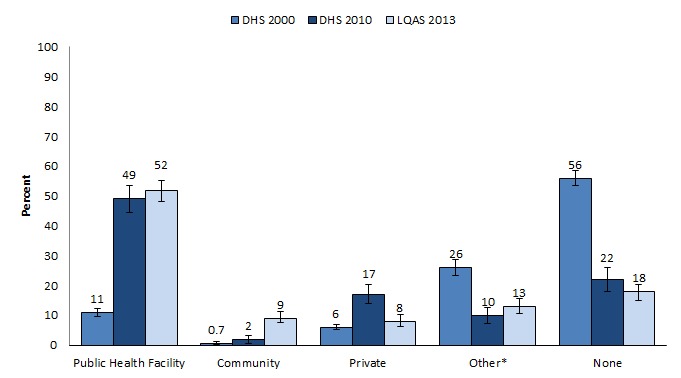
Place of treatment for fever, suspected pneumonia and diarrhoea in children under–5, 10 Catalytic Initiative districts. DHS – Demographic and Health Survey, LQAS – Lot Quality Assurance Survey. *Other – Private pharmacy, drug vendor/ store, shop, traditional healer, relative/friend.

The effect of the introduction of HSA delivered iCCM can be seen in the ‘place of treatment’ data. **Figure 4** shows a steady and significant increase in care–seeking for fever, suspected pneumonia and diarrhoea at community level (HSAs at village clinics) from less than 1% in 2000 to 9% in 2013 with a corresponding significant decrease in children receiving no care from 56% in 2000 to 18% in 2013.

Given the mortality declines and increases in coverage of critical interventions for child survival, the lives saved analysis predicted an under–5 mortality rate in the 10 CI districts of 84 per 1000 live births in 2013 when starting at a baseline mortality rate of 119 per 1000 live births in 2008.

**Figure 5:**
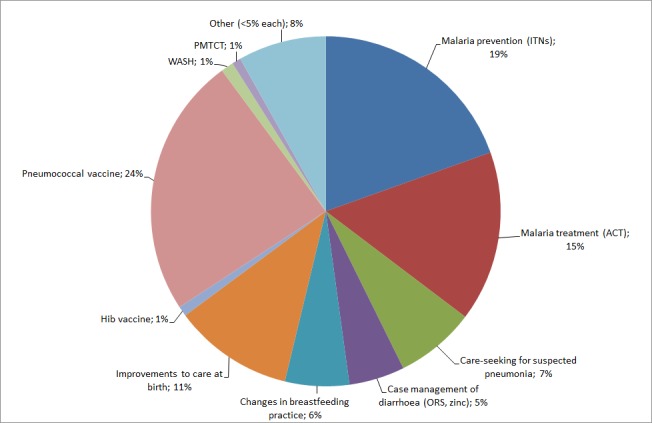
Percentage of child lives saved in Malawi (10 Catalytic Initiative districts), by intervention, in 2013, relative to a 2008 baseline. Improvements to care at birth include: labour and delivery management, antenatal corticosteroids for preterm labour, neonatal resuscitation, and clean birth practices. WASH indicators include improved water and sanitation and access to water connection in the home. ITNs – Percent of children <5 who slept under an Insecticide Treated Net the previous night; ACT – Artemisinin–combination therapy; ORS – Percentage of children <5 with diarrhoea in the last 2 weeks who received oral rehydration salts; PMTCT – Prevention of mother to child transmission of HIV; WASH – water, sanitation and hygiene; Hib – *Haemophilus influenzae* type B vaccine.

The proportion of child lives saved in 2013, by intervention, was calculated using the LiST estimation of 10 800 additional deaths averted in 2013 (relative to the 2008 baseline) as a denominator (**Figure 5**). The pneumococcal vaccine introduction in 2011, with rapid scale up given the existing immunisation platform was estimated to have averted one in four deaths and was the single largest contributor to lives saved in 2013 (24%, 2600 lives saved), followed by ITNs for households (19%, 2100 lives saved) and malaria treatment with artemisinin–combination therapies (15%, 1700 lives saved). Care seeking for suspected pneumonia contributed to 7% of lives saved in 2013 (800 lives saved for pneumonia) and case management of diarrhoea (ORS and zinc) contributed to 5% (400 lives saved for oral rehydration salts (ORS) and 150 for zinc for treatment of diarrhoea). Changes in breastfeeding practices contributed to approximately 6% of deaths averted (600 lives saved). Facility deliveries in the CI districts increased 29 percentage points (from 55% to 84%) in the CI period resulting in improvements in care at birth. These interventions accounted for 11% of all deaths averted even though this was not a direct focus of the CI programme. Of the remaining interventions, no single intervention saved more than 3% of child lives in 2013 (**Figure 5**). Stunting and wasting rates did not decline resulting in no measurable mortality reduction from interventions to address stunting and wasting. When comparing the pre–CI and CI periods, the proportion of deaths averted pre–CI was 15% in 2007 compared to baseline mortality in 2000, whereas during the CI period, the proportion was nearly double at 30% in 2013 compared to 2008 baseline mortality.

Major policies and programmes related to child survival were initiated in Malawi between 2004 and 2011 (**Figure 2**). The Essential Health Package in 2004 prioritised and strengthened community participation and delivery of free community health services through a set of evidence–based high impact interventions for children and adults. In 2005 the Reach Every District strategy was adopted to increase access to these interventions. In 2006, the Government of Malawi, together with UNICEF, WHO and the World Bank launched the Accelerated Child Survival and Development (ACSD) strategy which is an integrated approach based on the IMCI 5–year strategic plan 2006–2011 [25]. Implementation of the CI programme began in 2007 in support of the ACSD policy [8]. In 2008 guidelines for implementation of iCCM were developed and training of existing HSAs began across the country.

The desk review identified improvements in several macroeconomic indicators (**Table 2**). Between 2000 and 2012 there were noticeable changes in the per capita GDP which increased by $ 110 per capita (in constant 2011 international dollars) from $ 639 in 2000 to $ 749 in 2012) [2]. Per capita total expenditure on health increased from $ 9 in 2000 to $ 24 in 2012. Although external funding for health had been declining since 2006, it remained substantial at 53% of total expenditure for health in 2012, and more than double what it was in 2000 (26%). The poverty headcount (<$ 1.90/ day) declined from 74% in 2004 to 71% in 2010 [2].

**Table 2:** Comparison of broader health system and non–health system changes between 2000 and 2012 that might be expected to affect child survival

	Year			
Indicator	**2000**	**2006**	**2010**	**2012**
Gross domestic product per capita (PPP, constant 2011 international $)*	639	612	749	749
Total fertility rate	6.2	5.8	5.6	5.4
Female completion of lower secondary school	16% (1999)	13%	12%	12%
Per capita total expenditure on health	$9	$21	$30	$24
Total government expenditure on health (% of GDP)	6%	9%	8%	9%
External resources for health (% of total expenditure)	26%	59%	55%	53%
HIV prevalence (15–49 years)	15.8%	12.9%	11.2%	10.8%
Poverty headcount ratio @<$1.90 a day (2011 PPP)†	64% (1997)	74% (2004)	71%	

*GDP per capita based on purchasing power parity is gross domestic product converted to international dollars using purchasing power parity rates. An international dollar has the same purchasing power over GDP as the US dollar has in the United States. Data are in constant 2011 international dollars. Source: World Bank database [2].

†Poverty headcount ratio at $1.90 a day is the percentage of the population living on less than $1.90 a day at 2011 international prices.

A major set–back occurred in 2011 due to a successive combination of the impact of the global economic crisis and the withdrawal of funding locally by bilateral and multilateral partners from the Sector Wide Approach (SWAp) [26]. These events led to the devaluation of the Malawi currency as well as severe shortages in foreign currency and fuel. With Malawi’s extreme dependence on foreign aid, the country’s health budget was substantially reduced in the second quarter of 2011. Development assistance to the SWAp has not resumed but direct funding of projects by partners continues.

## DISCUSSION

Our results show that Malawi, one of the least developed countries in the world, ranking 170 out of 187 on the Human Development Index in 2012 [27], has managed to reduce under–5 mortality in the 10 CI districts by 100 deaths per 1000 live births (a 46% reduction) between 1991 and 2010. This has occurred in the context of changes, mainly at the policy and budgetary level, including increases in funding from both the government and donors [28], which enabled a conducive environment for implementation of the nation’s ambitious child survival policies.

The coverage of many priority child health interventions increased significantly since 2000, particularly preventive interventions including IPTp, immunisations and use of ITNs, as well as health behaviours such as early and exclusive breastfeeding and care–seeking for suspected pneumonia. The health interventions that account for the majority of the deaths averted are delivered at village clinic level through both outreach and stimulating increased demand (eg, immunisation, ITN distribution, care–seeking behaviours and treatment coverage). A number of high impact interventions increased in coverage at a higher rate than prior to the CI implementation. A similar AAC in coverage between 2000 and 2010 was seen in all districts across the country, evidence of the Ministry’s approach of scaling up high impact interventions across the entire country.

The contextual analysis provides insight into the lack of difference in mortality and coverage change between the CI and non–CI districts. The implementation of the Strategic Plan for Child Survival and subsequent scale up of iCCM occurred simultaneously across the whole country supported by a collective of partners and donors allocated to each of the 28 districts with a partnership agreement to guide implementation. Although the 10 CI districts were the focus of DFATD support, other donors implemented the same packages of support in the remaining 18 districts.

Our findings suggest that several factors worked synergistically to achieve the decreases in child mortality in Malawi. First, these improvements have taken place within a context of strong child health policies and clear leadership of the Ministry of Health, which, although donor dependent, channelled support in a co–ordinated manner to effectively implement national child survival policies [10,29,30]. This finding reinforces previous results from analyses of success factors related to declines in neonatal mortality in Malawi [13,31]. Second, the implementation of these policies and high impact interventions has been made possible through substantial external investments in the health system, particularly the contribution of donors. The official development assistance to maternal, child and newborn health in Malawi increased from US$ 51.7 million in 2003 to US$ 154.7 million in 2012 [28]. Third, investments in the capacity of the health work force has plausibly led to improvements in important health behaviours such as use of ITNs, facility deliveries, immunisation coverage, care–seeking and treatment for common childhood illnesses. These investments include strengthening, over decades, a dense network of over 10 000 HSAs nationally as well as the Emergency Human Resource Programme launched in 2004 leading to a doubling of professional health workers (including doctors, clinical officers and nurses) [32].

While HSAs play a very important role in the health system, the contribution that community level care (through HSAs) has made to child survival should not be overstated. While the 7 percentage point increase in care–seeking at the community level is substantial in a context where care–seeking at this level was 2% at the start of iCCM scale–up and almost non–existent prior to this, it does point to a need for greater efforts to generate demand for community care–seeking. However, it should be noted that there is a theoretical ceiling for treatment of childhood illness at community level since HSAs delivering iCCM services are situated in hard–to–reach areas and therefore not all communities sampled in household surveys will have been exposed to iCCM–trained HSAs. With just over a third of HSAs trained in iCCM, the Government of Malawi and its partners have yet to fully leverage the potential of this service delivery platform. However, this task–shifting needs to be aligned with investments to enable effective supervision and mentorship of this front–line cadre [33,34].

A strength of this evaluation is that the 10 CI districts are geographically–dispersed across all three regions of the country, thus limiting regional biases. Population–based household survey data on mortality and coverage from all 10 CI districts were available for analysis from the time of implementation of the CI (2006), during the CI period (sampling in 2010), and at the end of the programme (2013). These data were triangulated with an in–country document review to gain a more in depth understanding of the reasons for the quantitative changes in coverage and mortality.

There were weaknesses to this evaluation. First, the lack of oversampling to provide district level coverage estimates for all districts in the 2000 DHS. Although the data were aggregated across the two strata (CI and non–CI districts), there could be regional biases from the pre–CI estimates. A sensitivity analysis revealed no significant changes to the AAC comparisons (Table s8 in **Online Supplementary Document(Online Supplementary Document)**). Second, due to the mix of methods used to collect contextual data, we are unable to quantitatively estimate the relative contributions of wider changes in the health system and beyond to the reduction of child mortality. However we have followed the approach used in two previous country case studies [35,36], one of which is part of the Countdown to 2015 multi–institutional, multi–agency collaboration to track progress towards MDG goals 4 and 5 [36]. This case study will therefore add to the body of literature describing unique national pathways towards improved child survival.

Third, while the LiST model predicted within a margin of the measured mortality change, all factors must be applied when directly linking measured mortality reduction with coverage change. For this reason the results of the LiST analysis should be treated with caution. For example, factors outside of the health sector could have contributed to mortality declines, and/or incorrect assumptions could have been used for coverage of high impact interventions without empirical data available (eg, Kangaroo Mother Care) to run the LiST model.

Malawi has achieved remarkable progress in reducing child mortality and a recently released household survey reports a further decline which will almost certainly enable the country to achieve MDG4 [37]. However, one also needs to consider whether this trend is sustainable given Malawi’s heavy reliance on donor funding for the health sector and the risk of shifting global priorities. Malawi provides a strong example for countries in sub–Saharan Africa of how high impact child health interventions implemented within a decentralised health system with an established community–based delivery platform, can lead to significant reductions in child mortality. This example also highlights the imperative for the international community to commit to longer–term investments in health system strengthening and development as countries move from the “quick wins” related to survival to the more complex goals related to overall child health and development.
