# Supplementary material for: Assessment of Malawi’s success in child mortality reduction through the lens of the Catalytic Initiative Integrated Health Systems Strengthening programme: Retrospective evaluation
Source: J Glob Health. 2015 Nov 15;5(2):020412. doi: 10.7189/jogh.05.020412 (PMC4652924; doi:10.7189/jogh.05.020412)
Supplement: Online Supplementary Document [file jogh-05-020412-s001.pdf]

Online Supplementary Document

Assessment of Malawi's success in child mortality reduction through the lens of the Catalytic Initiative Integrated Health Systems Strengthening programme: Retrospective evaluation

J Glob Health 2015;5:020412

Table of contents

A. Additional details for the methods section 2
B. Alignment of indicator definitions 7
C. Mortality analysis 7
D. Evaluation of factors that may be associated with under-five mortality in Malawi 11
E. Additional details regarding the LiST analysis 12
F. Calculation of average annual change (AAC) in coverage 15
G. Sensitivity analysis 16

A. Additional details for the methods section

The 2000 and 2004 DHS were designed to provide estimates of health and demographic indicators at the national and regional levels with a subset of districts designated for oversampling to provide estimates at this level [1, 2]. In 2000 11 districts were oversampled and in 2004 10 districts were oversampled. The 2006 MICS was used instead of the 2004 DHS for the coverage analysis because the 2006 MICS had complete data for all 28 districts, a much larger sample size, and was closer to the baseline year of CI implementation in 2007.

Table s1: Description of data for mortality and coverage analysis

Survey and year	Main objectives of the survey	Sampling method	Sample sizes	Sample coverage	Dates of interview	Component of the questionnaire
DHS						
2000	Estimate socio-economic, demographic and health indicators at national, regional level and selected large districts which were over sampled	Stratified 2-stage cluster sampling; systematic sampling of clusters; systematic sampling of households	560 clusters, 14213 households, 13220 women 15-49	National sample	July – November 2000	Household questionnaire; women 15-49
2004	Estimate socio-economic, demographic and health indicators at national, regional level and selected large districts which were over sampled	Stratified 2-stage cluster sampling; systematic sampling of clusters; systematic sampling of households	522 clusters, 13664 households, 11698 women 15-49	National sample	October 2004 – January 2005	Household questionnaire; women 15-49
2010	Estimate socio-economic, demographic and health indicators at national, regional and district levels	Stratified 2-stage cluster sampling; systematic sampling of clusters; systematic sampling of	849 clusters, 24825 households, 23020 women 15-49	National sample	June – November 2010	Household questionnaire; women 15-49

		households				
MICS						
2006	Estimate socio-economic, demographic and health indicators at national, regional and district levels	Stratified 2-stage cluster sampling; systematic sampling of clusters; systematic sampling of households	1040 clusters, 30553 households, 26259 women 15-49	National sample	July – November 2006	Household questionnaire; women 15-49; children under 5
LQAS						
2013	Estimate demographic and health indicators in the 10 CI districts	Stratified 2-stage random sampling: random sampling of villages: random sampling of households	Sample of 1,273 respondents for each of the 6 universes (target groups) included in this analysis:<br>1) Mothers of children 0-5 months<br>2) Mothers of children 0-59 months<br>3) Mothers of children 0-59 months with diarrhea<br>4) Mothers of children 0-59 months with fever<br>5) Mothers of children 0-59 months with suspected pneumonia<br>6) Mothers of children 12-23 months	10 CI districts	November 2012 – May 2013	1) Mothers of children 0-5 months<br>2) Mothers of children 0-59 months<br>3) Mothers of children 0-59 months with diarrhoea<br>4) Mothers of children 0-59 months with fever<br>5) Mothers of children 0-59 months with suspected pneumonia<br>6) Mothers of children 12-23

						months

Panel s1 describes the data sources for the contextual analysis which were used to develop the policy and implementation timeline (figure 2 in the paper).

Panel s1: Data sources for the contextual analysis

Product type	Process of analysis / assessment
Documents (policies, programme reports, research reports and papers)	
• Policy and strategic plan documents of the Ministry of Health (IMCI strategic plan 2006-2011, Human Resources for Health Strategic Plan 2012-2016)<br>• Catalytic Initiative Support to Integrated Health Systems Strengthening annual progress reports prepared by UNICEF for the Department of Foreign Affairs, Trade and Development (DFATD) Canada.<br>• Programme and research reports from implementation partners (Population Services International, USAID, Save the Children, Management Sciences for Health, Johns Hopkins University)<br>• Peer reviewed articles on child survival in Malawi	• Review of policies and extraction of information on pivotal events and policy changes related to child survival<br>• Review and extraction of key implementation milestones<br>• Review of documents for information on scope of activities of partners, geographic areas covered, periods of implementation<br>• Pubmed search of literature on child survival in Malawi since 2004. Review of articles and extraction of information
Extraction of socio-political data from databases	
• Extraction of Macro-economic indicators (GDP, expenditure on health)<br>• Extraction of health indicators known to impact on child survival (fertility rate, HIV prevalence)<br>• Extraction of socio-economic indicators (female education, poverty headcount)	• Data extracted from World Bank Malawi country dataset and verified against DHS surveys for data collected from that source. Spreadsheet compiled with data from 2000 to 2012 for all selected indicators

B. Alignment of indicator definitions

The following adjustments were made to some indicators in order to align definitions between surveys: the denominator and numerator for tetanus vaccination and IPT/Fansidar during pregnancy which had been calculated over a 5-year period in DHS, have been restricted to live births in the two years preceding the survey to correspond with MICS definitions: "pharmacy" has been excluded from care-seeking providers in both DHS and MICS data for fever and suspected pneumonia. Vitamin A has been restricted from 6-59 months age group in MICS/DHS to 12-23 months in order to align it with the LQAS.

Some differences remained for the following indicators in the LQAS survey due to the restrictive data collection method used; data for IPT/Fansidar, early breastfeeding and tetanus toxoid during pregnancy were collected from mothers of 0-5 month old infants yet in the other surveys all mothers with a live birth in the past two years were considered. It was not possible to create a consistent definition across surveys because of lack of infant age within the MICS data file providing these three indicators from interviewed mothers/primary caregivers.

C. Mortality analysis

<u>Method of computation of under-five mortality rates</u>

Under-five mortality rates were computed for successive five year periods preceding the 2000, 2004 and 2010 DHS and the 2006 MICS using direct methods based on complete birth histories as recommended by the DHS statistical guide [3]. For each five-year period, age-specific mortality probabilities were computed for eight age groups: 0, 1-2, 3-5, 6-11, 12-23, 24-35, 36-47, 48-59 month using a synthetic cohort concept. This approach involves approximations of the number of children who enter an age range during a specific period. The resulting survival probabilities of death for each age interval were chained together to estimate the survival probability at age five, which was then converted into the under-five mortality rate.

The death rates in this paper are calculated using the following formula:

The estimated probability of death in age-interval *I in period j*:

Pr(j*i*) = $\boldsymbol{dji}/\boldsymbol{nji}$

where dj_i is the total number of deaths in period j for age-group *i* in 0, 1-2, 3-5, 6-11, 12-23, 24-35, 36-47, 48-59 months and nj_i is the total number of at risk.

The Life Table method then calculates survival probability as the product of 1 minus the conditional probability of death of all age-intervals as follows:

S(ji) **=** $\prod_{i=0}^{i=48-60}(\mathbf{1}-\mathbf{Pr}(\boldsymbol{ji}))$ for all *i* in 0, 1-2, 3-5, 6-11, 12-23, 24-35, 36-47, 48-59 months.

Therefore Under 5 mortality rate = **(1- *Sj(i)*) x 1000**

The standard errors for the computed under-five mortality were obtained using Jackknife repeated replications procedures [4].

Calculation of average annual change (AAC) in mortality

The Average Annual Change in mortality between year 1991 and 2010 was calculated on the log scale assuming linear change as per the below formula:

$$\boldsymbol{r}=\left(\boldsymbol{ln}\left(\frac{\boldsymbol{m}_{\mathbf{2010}}}{\boldsymbol{m}_{\mathbf{1991}}}\right)\right)\Big/(\mathbf{2010}-\mathbf{1991})$$

Tables s2a, b and c below give under-five mortality estimates for 5 year periods preceding the 2010 DHS with 95% confidence intervals for national, CI districts and non-CI districts. Data is from the Malawi 2010 DHS.

Table s2a: National under-five mortality rates based on 5-year periods preceding the 2010 MDHS, with the associated 95% confidence intervals.

5 year periods prior to the 2010 DHS	Estimate	95% confidence interval	
		LL	UL
1981-1985	256	166	346
1986-1990	246	195	297
1991-1995	216	178	254
1996-2000	180	160	200
2001-2005	145	130	159
2006-2010	112	98	126

Table s2b: Under-five mortality rates in the 10 CI districts* based on 5-year periods preceding the 2010 MDHS, with the associated 95% confidence intervals.

5 year periods prior to the 2010 DHS	Estimate	95% confidence interval	
		LL	UL
1981-1985	284	223	344
1986-1990	257	191	324
1991-1995	219	189	249
1996-2000	189	159	218
2001-2005	144	128	160
2006-2010	119	105	132

**Includes Karonga, Mzimba, Kasungu, Lilongwe, Dedza, Ntcheu, Balaka, Chiradzulu, Phalombe, Nsanje*

Table s2c: Under-five mortality rates in the non-CI districts based on 5-year periods preceding the 2010 MDHS, with the associated 95% confidence intervals.**

5 year periods prior to the 2010 DHS	Estimate	95% confidence interval	
		LL	UL
1981-1985	231	140	322
1986-1990	236	190	282
1991-1995	213	163	262
1996-2000	173	155	191
2001-2005	145	127	164
2006-2010	107	88	125

***Includes Blantyre, Chikwawa, Chitipa, Dowa, Machinga, Mangochi, Mchinji, Mulanje, Mwanza, Neno, Nkhata Bay (combined with Likoma), Nkhotakota, Ntchisi, Rumphi, Salima, Thyolo, Zomba*

Figure s1 below shows the trend in under-five mortality (1966-2010) in 5-year periods using data from four national household surveys.

Figure s1: Trend of under-five mortality rate based on 5-year periods preceding four national household surveys: DHS 2000; 2004; MICS 2006 and DHS 2010.

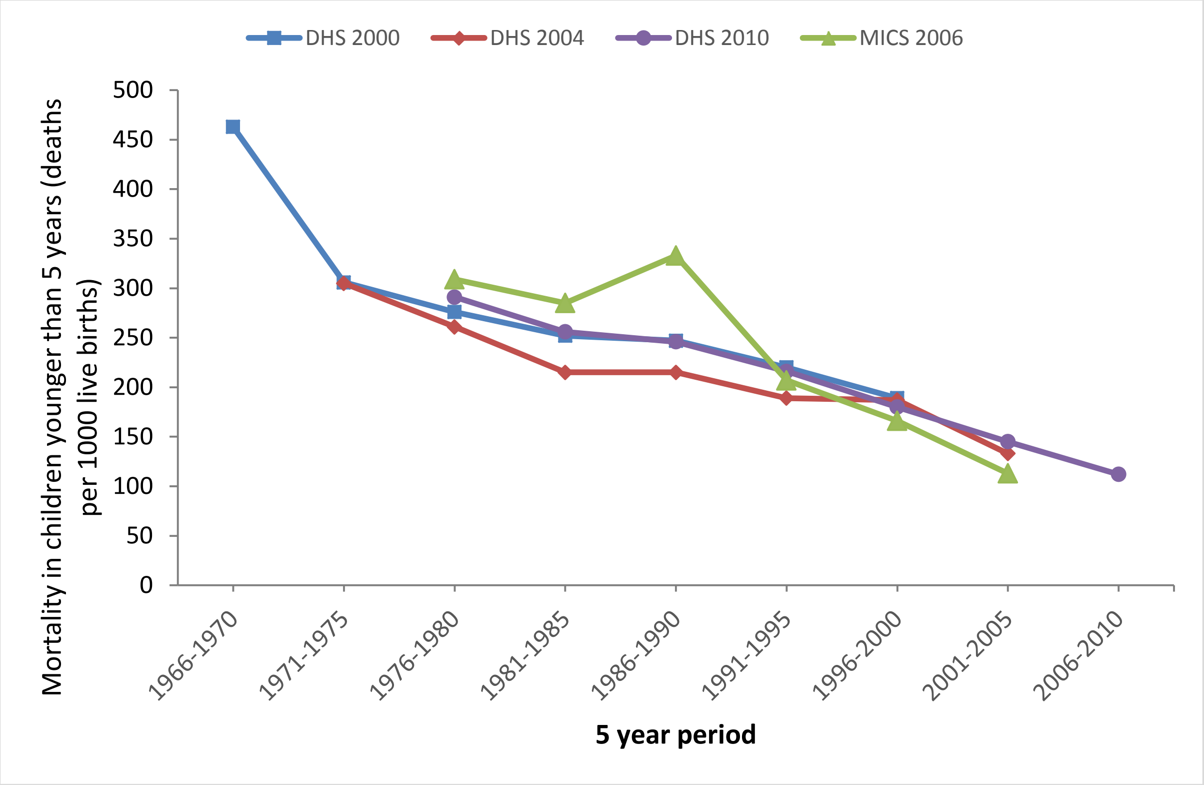

Dates on the x-axis represent 5-year periods preceding the surveys.

Table s3 below provides the under-five mortality rates for 5-year periods preceding four Malawi national household surveys.

Table s3: Trends in national under-five mortality rate and sample size from DHS 2000, 2004 and 2010 and MICS 2006, Malawi

	**U5MR (per 1000 live births)**			
**5 year periods prior to the surveys**	**DHS 2000**	**DHS 2004**	**MICS 2006**	**DHS 2010**
1966-1970	463			
1971-1975	306	305		
1976-1980	276	261	309	291
1981-1985	252	215	285	256
1986-1990	247	215	333	246
1991-1995	220	189	207	216
1996-2000	189	187	166	180
2001-2005		133	113	145
2006-2010				112
Sample size (household)	14213	13664	30553	23020

D. Evaluation of factors that may be associated with under-five mortality in Malawi

We assessed associations between under-five mortality and a number of factors including coverage of key health interventions and macro-economic indicators using simple linear regression, where we took the logarithm of mortality rate as the outcome. Both the outcome and the factors were collected for the period 2000-2010. Factors considered included GDP per capita, per capita expenditure on health, external resources for health (% of total expenditure), HIV prevalence, total fertility rates, as well as skilled birth attendance, care-seeking for suspected pneumonia and insecticide treated bed-net coverage.

We used a series of univariate and multivariate linear regressions, and retained factors in the final model if the respective association (coefficient) was statistically significant at p-value < 0.05. All the factors individually were significantly associated with under-five mortality. However, the final multivariate model retained only per capita expenditure on health as an independent predictor (Table s4).

Table s4: Multivariate linear regression of factors associated with under-five mortality reduction

**Factor**	**Coefficient**	**95% confidence interval**	***P* value**
**per capita expenditure on health (current US$)**	-0.01	-0.02 – -0.01	0.03

The model showed an inverse association between per capita expenditure on health and under-five mortality change (R-squared = 0.974). However this finding should be interpreted with caution due to the absence of sufficient data on other important contextual factors related to under-five mortality. It is plausible that the change in under-five mortality observed is due to a complex interplay of different factors, which cannot be causally linked statistically to the decline in mortality.

E. Additional details regarding the LiST analysis

The LiST analysis for the 10 CI districts combined population data and used an average of mortality, fertility and coverage data. Table s5 shows the data sources used for the baseline characteristics - population, fertility, mortality rates, causes of death and nutrition – for the Malawi LiST analyses. These were modified when necessary to best reflect the annual population and births in the 10 CI districts. Data for the 10 CI districts was aggregated and placed into one projection file. Coverage data for key indicators, representing the scope of the CI programme as well as broader health system indicators, were extracted from all of the available household surveys and other datasets for each time point available and entered into the projections. When coverage data for immunizations was not available from national surveys (e.g. for Hib, Hep B, and pneumococcal conjugate vaccine), WHO/UNICEF coverage estimates were used. Data was interpolated between surveys. Table s6 provides coverage inputs and data sources used in the LiST analysis.

Table s5: Additional data used to create LiST projections

Indicator	Source
Population served	National Statistics Office Malawi
Total fertility rate	DHS 2000 and 2010 and MICS 2006
Stunting, wasting rates	DHS 2000 and 2010 and MICS 2006
Cause of death	CHERG trend analysis
Mortality (under-5, infant, neonatal)	DHS 2000 and 2010 and MICS 2006

Table s6: Coverage data (%) input and data sources used for LiST analysis

	2000	2008	2013	Data source*
Pregnancy				
Antenatal care (4+ visits)	56.7	30.9	41.4	Malawi household surveys
Tetanus toxoid vaccination (protected at birth)	84	88	88	
IPTp for malaria prevention	29.7	58.2	84	Malawi household surveys
PMTCT<br>Single dose nevirapine<br>Dual ARV<br>ART started before current pregnancy<br>ART started during current pregnancy	0<br>0<br>0<br>0	 53<br>39<br>0<br>8	 5<br>34<br>23<br>39	LiST projection pre-entered data applied to subregional population
Childbirth				
Skilled birth attendance	56.6	60.3	76.2	Malawi household surveys
Facility birth	56.6	60.3	76.2	Malawi household surveys
Interventions within package:				*Intervention coverage calculated using default assumptions in LiST relating to facility births*
Clean birth practices	*47.1*	*51.1*	*64.6*	
Immediate assessment and stimulation	*43.6*	*47.3*	*59.8*	
Labor and delivery management	*55.5*	*60.3*	*76.2*	
Neonatal resuscitation	*25.0*	*25.1*	*34.3*	
Antenatal corticosteroids for preterm labor	*41.5*	*47.4*	*57.0*	
Antibiotics for pPRoM	*41.5*	*47.4*	*57.0*	
MgSO4 management of eclampsia	*41.5*	*47.4*	*57.0*	
Active management of the third stage of labor	*41.5*	*47.4*	*57.0*	
Induction of labor for pregnancies lasting 41+ weeks	*6.7*	*7.2*	*9.1*	
Breastfeeding				
Exclusive breastfeeding prevalence <1 month	85.9	90.2	94.2	Malawi household surveys
Exclusive breastfeeding prevalence 1-5 months	40.1	60.2	68.2	Malawi household surveys
Any breastfeeding 6-11 months	99.4	98.3	98.2	Malawi household surveys
Any breastfeeding 12-23 months	89.6	89.7	92	Malawi household surveys
Preventive				
Preventive postnatal care	10.9	27.7	41.4	Malawi household surveys
Complementary feeding	18.5	18.5	18.5	Malawi household surveys
Vitamin A supplementation	65.9	69.6	56	Malawi household surveys
Zinc supplementation	0	0	24.5	Malawi household surveys
Improved water source	63.4	75.8	77.4	
Water connection in the home	19.3	4.7	8.1	Malawi household surveys
Improved sanitation - Utilization of latrines or toilets	45.5	50.5	52.9	Malawi household surveys
Hand washing with soap	17	17	17	LiST projection pre-entered data
Hygienic disposal of children's stools	78.2	64	64	Malawi household surveys

Ownership of insecticide treated nets (ITN/LLIN)	13.8	54.1	93.8	Malawi household surveys
Vaccines				
BCG	92	95.0	95.0	Malawi household surveys
Polio	76	85.6	87.0	Malawi household surveys
DPT	82	86.6	88.0	Malawi household surveys
H. influenzae b	0	86.6	88.0	Malawi household surveys
HepB	0	86.6	88.0	Malawi household surveys
Pneumococcal	0	0	89.0	WHO-UNICEF immunization database
Rotavirus	0	0	81.0	WHO-UNICEF immunization database
Measles	81	82.7	87.0	Malawi household surveys
Curative				
Case management of premature babies	10.9	20.1	25.9	Calculated using facility birth data from household surveys
Thermal care	10.9	10.9	10.9	LiST projection pre-entered data
KMC - Kangaroo mother care	0	9.2	15.0	Estimated based on programme data provided by Save the Children
Full supportive care for prematurity	0	0.0	0.0	LiST projection pre-entered data
Case management of severe neonatal infection	31.4	34.3	40.2	Calculated using facility birth data from household surveys
Full supportive care for sepsis/pneumonia	25.5	28.4	34.3	Calculated using facility birth data from household surveys
ORS - oral rehydration solution	47	53.1	61.0	Malawi household surveys
Antibiotics - for treatment of dysentery	18.1	18.5	19.6	Malawi household surveys
Zinc - for treatment of diarrhea	0	0.0	24.5	Malawi household surveys
Oral antibiotics : case management of pneumonia in children	26.1	59.4	78.0	Malawi household surveys
Vitamin A - for treatment of measles	65.9	69.6	56.0	Malawi household surveys
Antimalarials - Artemesinin compounds for malaria	0	15.2	53.0	Malawi household surveys
ART	0	0	50.1	LiST projection pre-entered data applied to subregional population

* Malawi household surveys considered included DHS 2000, MICS 2006, DHS 2010, LQAS 2013

F. Calculation of average annual change (AAC) in coverage

The Average Annual Change in coverage was calculated on the log scale assuming linear change as per the below formula:

ln(Coverage(t1)/coverage(t0))/years, where years is the time period between surveys conducted at periods t1 and t0

Average annual change (AAC) in intervention coverage in CI and non CI districts

Table s7 below shows AAC in coverage for 10 indicators in the CI districts and the non-CI districts between 2000 and 2010.

Table s7: Average annual change in coverage for 10 indicators in CI and non-CI districts

Indicator	AAC 2000 – 2010, CI districts (n=10)	AAC 2000 – 2010, non-CI districts (n=16*)	p value
Tetanus toxoid vaccination of pregnant women	1.1% (-0.5% - 2.7%)	-0.1% (-2.0% - 1.8%)	0.32
IPTp	6.1% (4.1% - 8.1%)	8.1% (6.3% - 9.9%)	0.12
Early breastfeeding	7.9% (3.5% - 12.4%)	7.8% (4.4% - 11.2%)	0.96
EBF	5.7% (1.3% - 10.2%)	5.1% (2.2% - 8.0%)	0.81
Vitamin A supplementation	4.3% (1.8% - 6.9%)	4.5% (2.5% - 6.5%)	0.89
DPT3 vaccination	2.3% (0.9% - 3.7%)	1.9% (1.1% - 2.8%)	0.66
Measles vaccination	2.4% (0.9% - 3.9%)	1.9% (1.2% - 2.6%)	0.53
Care-seeking for suspected pneumonia	10.0% (2.3% - 17.8%)	7.9% (5.8% - 10.2%)	0.58
ITNs	18.0% (11.7% - 24.3%)	26.2% (17.6% - 34.8%)	0.11
ORS	6.8% (4.7% - 8.9%)	7.4% (4.6% - 10.2%)	0.72

IPTp = intermittent preventive treatment of malaria for pregnant women; ITNs = Insecticide Treated Nets; DPT = diphtheria, pertussis and tetanus; ORS = oral rehydration salts

**Neno district only has data available for one year (2010), estimates for Likoma district are merged with Nkhata Bay in all surveys due to the small size of Likoma.*

G. Sensitivity analysis

A sensitivity analysis was performed to determine the effect of oversampling in 11 of 28 districts in the 2000 DHS. The effect of removing the districts with no estimates in 2000 from the 2010 estimates resulted in no significant changes to the average annual change comparisons (table s8).

Table s8: Average annual change in coverage for 10 indicators in CI and non-CI districts within the oversampled districts alone.

Indicator	AAC 2000 – 2010, CI districts (n=4)	AAC 2000 – 2010, non-CI districts (n=7)	p value
Tetanus toxoid vaccination of pregnant women	0.002% (-2.1% -2.1%)	2.1% (0.7% - 3.6%)	0.05
IPTp	6.4% (5.6%-7.3%)	6.8% (5.4%-8.1%)	0.67
Early breastfeeding	3.1% (0.3% - 5.9%)	2.5% (1.8%-3.1%)	0.45
EBF	6.7% (4.2% -9.2%)	1.7%(-1.4%-4.8%)	0.02*
Vitamin A supplementation	1.2% (0.04%-2.3%)	2.0% (0.5%-3.4%)	0.38
DPT3 vaccination	1.1% (0.6%-1.7%)	1.0% (0.3%-1.7%)	0.68
Measles vaccination	1.1% (-1.2%-3.4%)	1.0% (0.4%-1.6%)	0.92
Care-seeking for suspected pneumonia	11.4% (2.5%-20.3%)	8.2% (4.2%-12.2%)	0.32
ITNs	25.2% (20.7%-29.7%)	31.2% (14.7%-47.6%)	0.53
ORS	5.3% (1.9% - 8.7%)	2.7% (0.5%-4.9%)	0.11

**Even though the p-value is <0.05, the confidence intervals overlap indicating low precision and hence not a strong difference between the two groups.*

References

1. National Statistical Office Malawi, ORC Macro: Malawi Demographic and Health Survey 2000. Zomba, Malawi and Calverton, Maryland, USA.: National Statistical Office and ORC Macro; 2001.
2. National Statistical Office (NSO) [Malawi] and ORC Macro: Malawi Demographic and Health Survey 2004. *Calverton, MD: National Statistical Office (Malawi) and ORC Macro.* Zomba Calverton; 2005: 330.
3. Rutstein S, Rojas G: Guide to DHS Statistics. Calverton: ORC Macro; 2006.
4. Pedersen J, Liu J: Child mortality estimation: appropriate time periods for child mortality estimates from full birth histories. PLoS Med 2012, 9(8):e1001289.
